# Changes in Pupil Area during Low-energy Femtosecond Laser-assisted Cataract Surgery

**DOI:** 10.18502/jovr.v14i3.4780

**Published:** 2019-07-18

**Authors:** Alireza Mirshahi, Katharina A. Ponto

**Affiliations:** ^1^ Dardenne Eye Hospital, Bonn, Germany; ^2^ Department of Ophthalmology, University Medical Center Mainz, Germany; ^3^ Center for Thrombosis and Hemostasis, University Medical Center Mainz, Germany

**Keywords:** Cataract Surgery, Femtosecond Laser, Pupil Size, Safety

## Abstract

**Purpose:**

To study the potential changes in pupil area within low-energy femtosecond-laser assisted cataract surgery (FLACS).

**Methods:**

A retrospective assessment of the pupil size was performed in the eyes undergoing FLACS using the Ziemer LDV Z8. We measured the pupil diameters as part of the images taken preoperatively and at the completion of laser pretreatment (after releasing the suction). We calculated the pupil area in 40 eyes of 40 patients (14 right and 26 left eyes). The mean ± standard deviation (SD) of age of the patients was 74 ± 7.4 years (range: 51-87). Paired *t*-test was used for statistical analyses. Subgroups were built with reference to age and preoperative pupil area (smaller than or equal to the median versus larger than the median).

**Results:**

The mean ± SD axial length, anterior chamber depth, white-to-white distance and lens thickness were 24.01 ± 1.47, 3.23 ± 0.4, 11.97 ± 0.49, and 4.59 ± 0.41 mm, respectively. The mean ± SD pupil area was 39.33 ± 7.1 mm${}^{2}$ preoperatively and 39.3 ± 6.75 mm${}^{2}$ after laser pretreatment. The mean ± SD change in pupil area was -0.03 ± 2.12 mm${}^{2}$. There were no statistically significant changes between preoperative and post-laser pupil areas (*P* = 0.93, 95% CI: -0.71 to 0.65). Comparisons within subgroups also did not detect pupil area reduction.

**Conclusion:**

This study did not detect statistically significant changes in pupil area after laser pretreatment using low-energy FLACS. This observation is in contrast to previous studies using other laser platforms.

##  INTRODUCTION

Femtosecond laser-assisted cataract surgery (FLACS) has undergone considerable evolution since its introduction by Nagy et al in 2009.^[1,2,3,4]^ Currently, FLACS is thought to be safe and effective, as reported by several studies.^[1,5,6]^ Nevertheless, several technology-specific complications and side effects have been reported with the use of FLACS.^[7,8]^ Intraoperative miosis has been repeatedly reported as a common problem in association with FLACS. The narrowing of the pupil after laser pretreatment makes surgery more challenging to the surgeon, potentially resulting in a higher rate of capsule-related complications.^[9]^ Previous studies using early “high-energy" femtosecond lasers have shown a substantial increase in the number and severity of episodes of intraoperative miosis with a prevalence ranging between 9.5% and 32%.^[7,10,11,12,13]^ In a previous study, Jun et al reported that the duration of laser pretreatment and patient age correlated with decreased pupil area measured by intraoperative surgical images.^[13]^ Researchers believe that increased levels of prostaglandin E2 (PGE2), as measured immediately after laser pretreatment, are responsible for intraoperative narrowing of the pupil.^[12,13,14,15]^ PGE2 is released when ocular tissue is exposed to femtosecond laser cutting side effects.^[16]^

Conventional “high-energy" femtosecond lasers emit pulses with an energy in the 4 to 15 microjoule (μJ) range,^[7,10,14]^ whereas the newer low-energy concept uses high-pulse repetition rates above 1 MHz and a low-pulse energy in the nanojoule range^[16]^ This is achieved using a high numerical aperture in the laser focusing optics,^[15]^ enabling small laser spot sizes.

We hypothesized that the number and extent of episodes of intraoperative miosis would decrease in FLACS using a low-energy femtosecond laser compared to previously published literature. Therefore, we conducted this study to assess the change in pupil size in eyes undergoing cataract surgery using a low-energy femtosecond laser.

##  METHODS

This retrospective case series included eyes that had undergone FLACS using a Ziemer Z8 Femto LDV (Ziemer Ophthalmic System, Port, Switzerland) and an Alcon Infinity phacoemulsification system (Alcon Lab., Fort Worth, TX, USA) in Dardenne Eye Hospital, Bonn, Germany. All surgeries were performed by one experienced surgeon (AM) between September 2016 and April 2017.

The following data were extracted from patient records: age, laterality of surgery, axial length, anterior chamber depth, lens thickness, white-to-white distance, and special notes in the surgery report. Data on axial length, anterior chamber depth, lens thickness, and white-to-white were taken from laser biometry performed on the day of surgery (IOLMaster 700, Carl Zeiss Meditec, Jena, Germany). If data of both eyes of a patient were available, records of the first surgical eye were used.

We used images taken from surgical videos at the following time points: (1) preoperatively, shortly before docking the laser and (2) immediately after vacuum suction released (end of femtosecond laser pretreatment). The ethics committee of the North Rhine Medical Chamber ruled that approval was not required for this retrospective study. It was performed in accordance with the tenets of the Declaration of Helsinki.

###  Surgical Technique

One experienced surgeon (AM) performed all femtosecond laser pretreatments and phacoemulsification procedures. All patients received the local standard-of-care preoperative pupil enlargement regimen consisting of tropicamide 5 mg/ml (Mydriaticum StullnⓇUD, Pharma Stulln GmbH, Stulln, Germany) and phenylephrine 5% (Neosynephrin-POSⓇ5%, Ursapharm Arzneimittel GmbH, Saarbrücken, Germany) eye drops, four times each. No patient received additional nonsteroidal anti-inflammatory drugs (NSAIDS). All surgeries were performed under peribulbar anesthesia. With the surgeon sitting at the 12 o'clock position, the Ziemer Femto LDV Z8 was positioned in an oblique angle. After disinfection and sterile draping, the femtosecond laser interface was positioned and vacuum suction was applied (approximately 420 mbar). Standard laser parameters were 6-mm-diameter laser lens fragmentation in six pieces, at 105% laser energy followed by a 5.2-mm-capsulotomy diameter using 90% laser energy. Suction was released after the completion of capsulotomy. No other surgical steps were done with the femtosecond laser system. The surgeon moved on with further surgical steps including posterior limbal main incision of 2.8 mm, two paracenteses of 1.1 mm each, introduction of a dispersive viscoelastic device into the anterior chamber, removal of capsulotomy by forceps, hydrodissection, hydrodelineation, high-vacuum phacoemulsification, bimanual removal of lens cortex, posterior capsule polishing, IOL implantation, bimanual removal of viscoelastic device, and hydration of the paracentesis.

###  Pupil Area Measurement

We used images taken from the surgical videos to measure the horizontal and vertical diameters of the pupil. Fiji, an image processing package of ImageJ software Version 2.0.0-rc-49/1.51a was used to measure the pupil diameters in pixels. The pixel measurements were then converted into millimeters, individually for each patient, using the constant limbus horizontal and vertical size as a reference. Assuming the pupil has an ellipsoid shape, we calculated pupil area as vertical radius multiplied by horizontal radius multiplied by π.

###  Statistical Analyses

As there is no previous data on this topic, the present study was done as a pilot project. Besides identification of potentially associated parameters, we aimed to establish baseline data to be used for a thorough sample size calculation in a future study. We calculated descriptive measurements for the pupil area at the time points mentioned earlier and the difference between preoperative and post-laser pupil areas. The main analysis examined possible differences in the pupil area of the individual measurements from the baseline preoperative measurement. To detect effects by larger or smaller preoperative pupil areas, a subgroup analysis was performed to evaluate the changes in pupil area in eyes with preoperative pupil areas smaller or larger than the median pupil size of all eyes included in the study. Furthermore, we separately evaluated eyes of older and younger patients (age ≤ the median age versus age > the median age). We used paired t-tests for statistical analyses. *P*-values < 0.05 were considered statistically significant. All statistical analyses were performed using SPSS (Statistical Package for the Social Sciences, version 25, Chicago, Illinois).

##  RESULTS

We included 40 eyes of 40 patients in this retrospective study (mean age: 74 ± 7.4 years, range: 51-87) with complete data available within the study period. If data were available from both eyes, data from the first eye operated on were used. The study sample comprised 14 (35%) right and 26 (65%) left eyes. Preoperatively, glaucoma and pseudoexfoliation were diagnosed in three eyes (7.5%). Further descriptive data, including axial length, anterior chamber depth, lens thickness, and white-to-white distance are illustrated in Table 1.

**Table 1:** Preoperative values of the relevant morphologic parameters in 40 consecutive eyes undergoing low-energy femtosecond-laser assisted cataract surgery


**Preoperative parameters**	**Mean ± Standard Deviation [mm]**	**Median [mm]**	**Minimum [mm]**	**Maximum [mm]**

Axial length	24.01 ± 1.47	24.02	21.34	27.13
Anterior chamber depth	3.23 ± 0.4	3.23	2.39	4.01
Lens thickness	4.59 ± 0.41	4.62	3.69	5.32
White-to-white distance	11.97 ± 0.49	12.0	10.8	12.8

**Figure 1:**
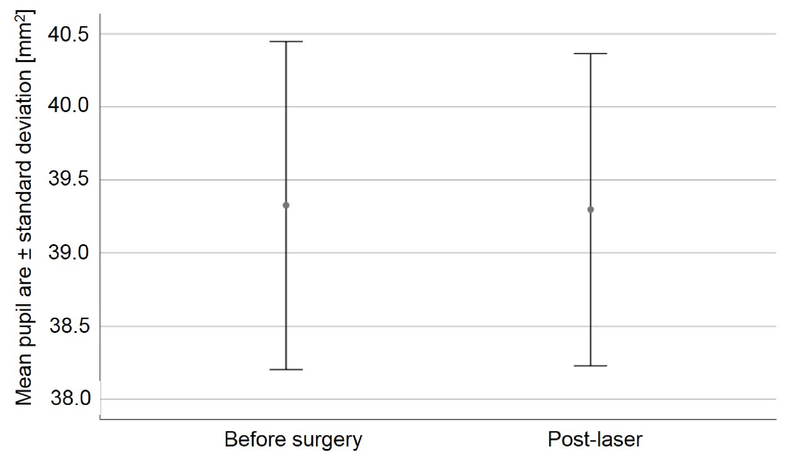
Preoperative and post-laser pupil areas in 40 consecutive and unselected eyes undergoing low-energy femtosecond-laser assisted cataract surgery (FLACS). Error bars showing the means ± standard deviations. Preoperatively, the mean pupil area was 39.33 ± 7.1 mm${}^{2}$. Immediately after the completion of laser pretreatment (after suction release), the mean pupil area was 39.3 ± 6.75 mm${}^{2}$. A paired t-test revealed no statistically significant changes between the preoperative and post-laser pupil areas (*P* = 0.93, 95% CI: -0.71 to 0.65).

Preoperatively, the mean, standard deviation, median, minimum and maximum values were 7.01 ± 0.65, 7.12, 5.51, and 8.27 mm, respectively, for horizontal pupil diameter, 7.09 ± 0.65, 7.09, 5.75, and 8.46 mm, respectively, for vertical pupil diameters, and 39.33 ±7.1, 39.61, 26.87, and 54.64 mm${}^{2}$, respectively, for the pupil area. The mean change between preoperative and post-laser pupil areas was -0.03 ± 2.12 mm${}^{2}$ (median: -0.35, minimum: -5.13, maximum: 4.16). Figure 1 illustrates preoperative and post-laser pupil areas. A paired t-test revealed no statistically significant changes between preoperative and post-laser pupil areas (*P* = 0.93, 95% CI: -0.71 to 0.65). In the subgroup of eyes with larger preoperative pupils (pupil area before surgery above the median of 39.61 mm${}^{2}$), the mean change of the pupil area after FLACS did not change significantly; in the group of eyes with a preoperative pupil area of 39.61 mm${}^{2}$ or smaller, it changed from 33.90 ± 4.41 mm${}^{2}$ preoperatively to 34.29 ± 5.00 mm${}^{2}$ after FLACS (95% CI: -0.58 to 1.35; *P* = 0.412); and in the group of eyes with a preoperative pupil area larger than 39.61 mm${}^{2}$, it changed from 44.75 ± 4.68 mm${}^{2}$ preoperatively to 44.31 ± 4.02 mm${}^{2}$ after FLACS (95% CI: -1.45 to 0.56; *P* = 0.367). In eyes of patients aged 75.5 years or younger, the mean change of pupil area after FLACS was 0.08 ± 1.88 mm${}^{2}$ (95% CI: -0.80 to 0.96; *P* = 0.858), and in eyes of patients older than the median age of 75.5 years, it was -0.13 ± 2.38 mm${}^{2}$ (95% CI: -1.25 to 0.98; *P* = 0.803).

##  DISCUSSION

To the best of our knowledge, this retrospective study is the first to assess pupil sizes of eyes undergoing low-energy FLACS. We could not detect any changes in the pupil area when comparing preoperative pupil status with post-laser size. This is in contrast to previous studies using other laser platforms that assessed pupil changes in high-energy FLACS. Diakonis et al compared the effect of three laser platforms (LenSx; Alcon Laboratories, Inc., Fort Worth, TX, Catalys; Abbott Medical Optics Inc., Santa Ana, CA, and Victus; and Bausch & Lomb, Inc., Rochester, NY) on pupil diameter^[12]^ and found a mean pupillary miosis of 1.42 ± 1.26 mm for the LenSx, of 0.66 ± 0.89 mm for the Catalys, and of 0.14 ± 0.34 mm for the Victus groups. Almost one-quarter of eyes included in this study demonstrated a pupil diameter of 6 mm or less. Jun et al report a 29.7% decrease in pupil area after femtosecond laser pretreatment in a study sample of 56 eyes.^[13]^ The same study group reported, in a follow-up comparative study, that the preoperative topical ketorolac tromethamine 0.45% significantly reduced femtosecond laser-associated miosis and inhibited prostaglandin E2 elevation in the aqueous humor.^[15]^

With those systems, a larger pupil diameter before FLACS was associated with greater miosis. This is, again, in contrast to the present study, as we were not able to detect a FLACS-induced miosis even in the subgroup of eyes with larger preoperative pupil areas. Similarly, we did not observe any changes when subdividing data into various age groups.

Our results are of clinical relevance because small pupil size is generally considered a challenge, potentially leading to a higher incidence or severity of complications in cataract surgery.^[9]^ Thus, we believe that the absence of femtosecond laser-associated intraoperative miosis using a low-energy platform may make surgery less challenging and less traumatizing.

Because pupils do not always have an exactly circular shape, measuring area changes are more accurate than considering only diameter in one dimension. Thus, we believe the most appropriate—and probably most sensitive—value to be assessed in similar studies is the calculated pupil area, rather than diameters. When reporting pupil diameters, both horizontal and vertical diameters should be considered.

The “low-energy" concept using a high numerical aperture in the femtosecond laser optics is thought to be a valuable evolutionary step forward toward smaller laser spots, thereby reducing collateral damage to the surrounding ocular tissue.^[17]^ While "high-energy" femtosecond lasers emit energy in the microjoule range, the modern low-energy concept combines high repetition rate above 1 MHz and pulse energies in the nanojoule range^[17]^ in order to achieve precise tissue cuts with minimal mechanical side effects. One possible explanation for the observation made in our study is that a low-energy laser platform probably produces lower "collateral damage" to the surrounding tissue, thus resulting in lower amounts of prostaglandins and, thereby, no (or negligible) intraoperative pupil narrowing. In fact, researchers could not detect meaningful increases in prostaglandin levels in the aqueous humor after low-energy FLACS, as reported in a preliminary clinical study (personal oral communication with Professor R. Menapace, May 2018). This finding is in line with our observation of unchanged pupil area. Furthermore, it supports the hypothesis that increased levels of prostaglandins are causative for intraoperative miosis in FLACS.^[16,18]^ Nevertheless, caution is warranted because those results have not yet been published in the peer-review literature. Another possible explanation for our observation of unchanged pupil area could be the time lapse between the femtosecond laser pretreatment and other surgical parts: When using the Ziemer Z8 laser, the surgery can be continued immediately after the completion of laser pretreatment, while in other lasers, the patient must be transferred to another surgical table or theatre. The larger short time lapse between laser pretreatment and other surgical steps may be another explanation for much higher incidences of intraoperative miosis in previous studies.^[13,14,18]^

FLACS requires the application of a suction device to stabilize the laser head and focus the laser beam accurately. This may cause a significant escalation IOP, which has been demonstrated for the Femto LDV Z8 in porcine eyes. Ebner et al showed that during the vacuum application of the liquid patient interface values were higher in the anterior chamber compared with the intravitreal pressure measurements.^[19]^ The higher predefined vacuum level (350 versus 420 mbar) resulted in significant higher intracameral IOP. Another porcine in vivo model showed that IOP with the Ziemer LDV femtosecond laser was lower using the liquid patient interface compared to the flat applanation system.^[20]^

As the present study was retrospective in design, no interventions besides those done within the clinical routine were performed. Therefore, we did not measure IOP during laser application. There are no previous studies on IOP in humans using the Femto LDV Z8. However, Schultz et al reported a minor increase in the IOP using the fluid-filled interface.^[21]^ Higher values have been reported in the literature with flat and curved applanating contact interfaces.^[21]^ At the same time, miosis with the Catalys has been reported.^[22]^ Therefore, we assume that it is not the IOP alone that contributes to miosis and that other factors like energy used might be more relevant.

The following limitations of our study merit consideration: (1) a relatively small sample size of 40 eyes; (2) the measurement of pupil sizes at only two time points; (3) the study sample was completely Caucasian; (4) no intraoperative measurement of prostaglandins in aqueous humor; and (5) a very short time lapse between laser pretreatment and further surgical steps. Future studies will need to shed light on these limitations. With this aim in mind, a thorough sample size estimation and power calculation are done on the basis of the present results and will be used for a future study that focuses mainly on the parameters found to be of potential interest in this baseline study. Nevertheless, we believe our study results are valuable, as unchanged pupil dimensions will make a cataract surgery less challenging.

##  Financial Support and Sponsorship

Alireza Mirshahi, MD is a consultant to Ziemer Ophthamic, Systems AG, Port, Switzerland; Katharina Ponto, MD was funded by the Federal Ministry of Education and Research (BMBF 01EO1003).

##  Conflicts of Interest

There are no conflicts of interest.
